# Eicosanoid production by *Candida parapsilosis* and other pathogenic yeasts

**DOI:** 10.1080/21505594.2018.1559674

**Published:** 2019-01-07

**Authors:** Tanmoy Chakraborty, Renáta Tóth, Attila Gácser

**Affiliations:** a Interdisciplinary Excellence Centre, Department of Microbiology, University of Szeged, Szeged, Hungary; b MTA-SZTE “Lendület” “Mycobiome” Research Group, University of Szeged, Szeged, Hungary

**Keywords:** *Candida parapsilosis*, pathogenic yeast, fungal eicosanoids, virulence

## Abstract

Eicosanoids are bioactive lipid mediators generated in almost all mammalian cells from the oxidation of arachidonic acid and other related twenty-carbon polyunsaturated fatty acids (PUFA). Eicosanoids regulate various physiological functions, including cellular homoeostasis and modulation of inflammatory responses in mammals. The mode of action of these lipid mediators depend on their binding to different G-protein coupled receptors. The three main enzymatic pathways associated with their production are the COX pathway, LOX pathway and cytochrome P450 pathway. Interestingly, investigations have also revealed that several human pathogenic fungi are capable of producing these bioactive lipid mediators; however, the exact biosynthetic pathways and their function in pathogenicity are not yet extensively characterized. The aim of the current review is to summarize the recent discoveries pertaining to eicosanoid production by human pathogenic yeasts with a special focus on the opportunistic human fungal pathogen *Candida parapsilosis*.

## Introduction

Oxylipins are oxidized lipid molecules generated from the oxidation of polyunsaturated fatty acids [1]. Eicosanoids are oxylipin molecules and the main precursor for their production is the twenty-carbon chain fatty acid molecule, arachidonic acid. Prostaglandins, thromboxanes, prostacyclins, leukotrienes, lipoxins, hepoxilins, hydroxy fatty acids, hydroxylated fatty acids and epoxy derivatives all belong to the eicosanoid family [2]. They are synthesized through enzymatic as well as non-enzymatic pathways (non-enzymatic free-radical-induced peroxidation of PUFAs) [3–5]. The majority of our knowledge available regarding eicosanoid biology derives from research performed on mammalian cells. Eicosanoids regulate various functions, mainly during inflammation and protective immune responses, and they also act as messengers in the central nervous system. Remarkably, they function as both pro-, as well as anti-inflammatory or pro-resolving mediators during immune responses against infections [3]. Although bioactive eicosanoid production by yeasts has been acknowledged since the early 1990’s [6], detailed descriptions of their biosynthetic pathways and function is still unavailable. Based upon the currently available studies, in this review, we provide an up-to-date and brief summary of eicosanoid production in pathogenic yeasts as well as their role in pathogenesis development, with a special focus on an emerging fungal pathogenic species, *Candida parapsilosis*.

### Eicosanoid production by human pathogenic yeasts

**Figure 1.**
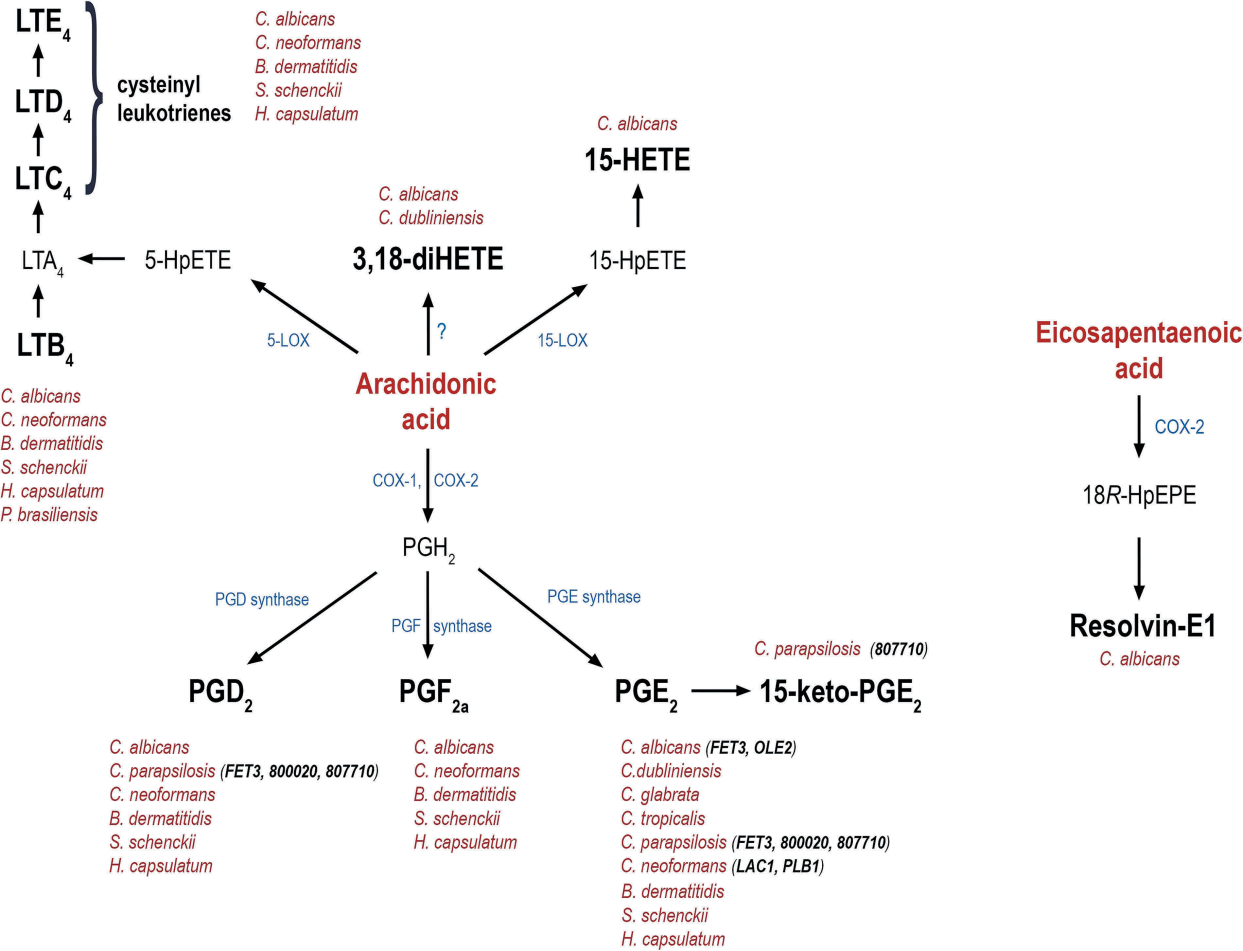
Schematic representation of eicosanoid biosynthesis, their production by fungi and the corresponding genes involved in their production. Eicosanoid production from the precursor arachidonic acid or eicosapentaenoic acid. Besides mammals, cysteinyl leukotrienes, 3,18-diHETE, 15-HETE, PGD_2_, PGF_2a_, PGE_2_, 15-keto-PGE_2_ and resolvin-E1 are also produced by different pathogenic fungi. Although the exact biosynthetic pathways remain unknown, several fungal genes have been proposed to regulate their synthesis. These include *FET3* and *OLE2* in *C. albicans, FET3 (CPAR2_603600), CPAR2_800020* and *CPAR2_807710* in C. *parapsilosis*, and *LAC1* and *PLB1* in *C. neoformans*.

In human pathogenic yeasts, the presence of fungal eicosanoids was first reported in the opportunistic fungal pathogen *Candida albicans*. In 2001, Deva et al. reported the production of 3,18-dihydroxy-5,8,11,14-eicosatetraenoic acid (3,18 di-HETE) by *C. albicans* from exogenous arachidonic acid, as determined by GC/MS analysis [7]. In the same year, Noverr et al. showed that both *C. albicans* and *Cryptococcus neoformans* were able to generate immunomodulatory prostaglandin from exogenous arachidonic acid [8,9]. The authors referred to this molecule as PGEx due to its cross-reactivity with the “E” class of prostaglandin in ELISA, although mass spectroscopic analysis later revealed that the identified prostaglandin was PGE_2_ [10]. Besides HETE and PGE_2_, these species are also able to produce PGD_2_ and PGF_2α_ as well as leukotrienes (LTB_4_, cysteinyl leukotrienes) from exogenous arachidonic acid [11]. Subsequently, *C. albicans* was also shown to produce the pro-resolving lipid mediator Resolvin E1 (RvE1), that is chemically identical to those produced by human cells and its biosynthetic precursors, 18-hydroxyeicosapentaenoic acid (HEPE), 15-HEPE and 5-HEPE [12]. In recent years, investigations have shown that non-*albicans Candida* species are also capable of producing immunomodulatory prostaglandins. These species include *C. dubliniensis, C. tropicalis* and *C. glabrata* [13,14]. Interestingly, *C. albicans* planktonic cells and biofilms are able to produce PGE_2_ from exogenous arachidonic acid [15–17] and the production of 15-HETE by *C. albicans* biofilm has also been reported [18]. It has also been reported that both the high and low virulent strains of the human pathogenic dimorphic fungus *Paracoccidioides brasiliensis* produce PGE_2_ and leukotriene B_4_ from the same substrate [19–21]. Pathogenic dimorphic fungi with an infectious yeast phase such as *Histoplasma capsulatum, Blastomyces dermatitidis* and *Sporothrix schenckii* can also produce a range of eicosanoids namely PGE_2_, PGD_2_, PGF_2α_ and leukotrienes from exogenous arachidonic acid [11]. Our current knowledge on eicosanoid production by pathogenic yeasts is summarized in Table 1. and Figure 1.

**Table 1.** Eicosanoids produced by human pathogenic yeasts and genes identified for PGE_2_ production in *C. albicans, C. parapsilosis* and *C. neoformans.*

Species	Eicosanoid	Genes identified for PGE2 production	References
*C. albicans*	3,18 di-HETE; 15-HETE; cysteinyl leukotrienes; LTB_4_; PGD_2_; PGE_2_; PGF_2α_; Resolvin1.	*FET3, OLE2*	7,9,10,11,17
*C. dubliniensis*	3,18 di-HETE, PGE_2_.		15
*C. glabrata*	PGE_2_		13
*C. tropicalis*	PGE_2_		13
*C. parapsilosis*	PGE_2_, PGD_2_, 15-k-PGE_2_	*CPAR2_603600, CPAR2_800020, CPAR2_807710*	44,45
*C. neoformans*	Cysteinyl leukotrienes; LTB_4_; PGD_2_; PGE_2_; PGF_2α_	*LAC1, PLB2*	8,10,24,25
*P. brasiliensis*	PGEx, LTB_4_		18, 19, 20
*B. dermatitidis*	PGE_2_, PGD_2_, PGF_2α_, CysLT,LTB_4_		10
*H. capsulatum*	PGE_2_, PGD_2_, PGF_2α_, CysLT, LTB_4_		10
*S. schenckii*	PGE_2_, PGD_2_, PGF_2α_, CysLT, LTB_4_		10

### Fungal eicosanoids in pathogenesis and immune regulation

Eicosanoid signaling regulates the mammalian immune system similarly to cytokine signaling [3]. They function during both the generation and the resolution of inflammatory reactions, and also participate in cellular homoeostasis [3] . Fungal infections can also induce the production of eicosanoids in different host cells, which contributes to the generation of an antifungal immune response [22–29]. Fungal eicosanoids modulate host immune responses as well as pathogenesis [30,31]. For example, the PGE_2_ produced by *C. albicans* induces yeast to hyphal transition, which is an important virulence trait of pathogenic fungi [32,33]. However, it has been shown previously that the null mutant strain of *FET3* did not alter pathogenicity compared with the wild-type strain in the mouse model of systemic candidiasis [34]. In contrast, *C. neoformans* deletion mutants of phospholipase B (*PLB*) or laccase (*LAC*) enzymes are less virulent in mice compared to the wild type strain, indicating the role of these genes in pathogenesis [33,^35^], albeit their functions impact more than eicosanoid biology. Fungal prostaglandins produced by these two species have also been confirmed to have immunomodulatory functions as they alter host cytokine responses by down-regulating chemokine (IL-8) and pro-inflammatory cytokine (e.g. TNFα) production, while up-regulating anti-inflammatory responses by promoting IL-10 release [8]. In the presence of human keratinocytes, *C. albicans, C. tropicalis* as well as *C. glabrata* produced 10-fold more PGE_2_ [14]. Taken together, these observations indicate the importance of fungal eicosanoids in host-pathogen interactions during fungal infection.

### Eicosanoid biosynthesis genes identified in human pathogenic yeasts

The three main enzymatic pathways involved in eicosanoid production in mammals include cyclooxygenases (COX), lipoxygenases (LOX) and cytochrome P450 enzymes [4]. *In silico* analysis of the recently available whole genome sequences of pathogenic fungi did not identify homologues of the corresponding mammalian genes. This indicated the presence of novel fungal eicosanoid biosynthetic pathways that may differ from the previously described mechanisms in mammals [36]. The use of different enzyme inhibitors against COX, such as acetylsalicylic acid (ASA) and other non-steroidal anti-inflammatory drugs (NSAIDs), as well as LOX inhibitors was inconclusive [8,12,22,23] as the addition of these inhibitors reduced eicosanoid production as well as concomitantly reducing the viability of the fungi.

After the discovery of prostaglandin molecules in *C. albicans*, two non-COX/LOX-related enzymes were reported to be involved in PGE_2_ production in this species: a fatty acid desaturase, Ole2p, and a multicopper oxidase, Fet3p [10]. The homozygous deletion mutant strains of the corresponding genes showed a significant reduction in PGE_2_ levels. The fact that PGE_2_ production was still detectable in both *ole2Δ/Δ* and *fet3Δ/Δ* strains, indicated the presence of additional enzymes that could also be involved in the biosynthesis of this eicosanoid. Using a specific inhibitor 6-(2-propargyloxyphenyl)hexanoic acid (PPOH) against cytochrome P450, the involvement of these enzymes was confirmed in PGE_2_ production in both *C. albicans* and *C. dubliniensis* biofilms [16]. The biosynthesis of RvE1 in *C. albicans* is also sensitive to lipoxygenase and cytochrome P450 monooxygenase inhibitors [12]. In *C. neoformans*, the *LAC1* laccase, another multicopper oxidase, was further identified as a regulator of PGE_2_ production, as the *lac1Δ/Δ* deletion mutant strain showed a reduction in PGE_2_ production. Additionally, the recombinant cryptococcal laccase enzyme is efficient in converting PGG_2_ to PGE_2_ but did not generate any new prostaglandins when incubated with only arachidonic acid or PGH_2_ [24]. The deletion of the *C. neoformans* phospholipase (*PLB1*) gene also resulted in a reduction in PGE_2_ production [25].

The inclusion of COX like enzymes in PGE_2_ biosynthesis has been implicated in *P. brasiliensis*, although the corresponding biosynthetic pathway is yet unexplored [19,20]. The significant reduction of LTB_4_ production by both selective or non-selective LOX inhibitors (MK886 or nordihydroguaiaretic acid) in *P. brasiliensis* indicated that the fungus produces LTB_4_ by using the LOX pathway or with a biochemically similar enzyme [21].

### Eicosanoid production by *Candida parapsilosis*

*Candida* species remain the most prevalent cause of invasive fungal infections, exceeding invasive aspergillosis and mucormycosis [37,38] and other infections by pathogenic fungi. Although, *C. albicans* is still the most common cause of invasive candidiasis, bloodstream infections caused by non-*albicans Candida* species such as *C. glabrata, C. krusei, C. auris, C. parapsilosis*, and *C. tropicalis*, altogether have risen to account for approximately one-half of all candidemia cases [39].*C. parapsilosis* is a commensal of the skin and it is also frequently isolated from the gastrointestinal tract [40]. This species is one of the major causes of invasive fungal infections in premature infants [41]. The incidence of *C. parapsilosis* is increasing in this particular patient group and it outnumbers *C. albicans* infections in some geographic regions [42]. *C. parapsilosis* is known for its ability to form biofilms on catheters and other implanted devices [43,44]. Different risk factors that are associated with *C. parapsilosis* driven neonatal candidiasis include low birth weight (<1500 g), prematurity, prior colonization, the use of parenteral nutrition, intravascular catheters and prolonged treatment with antibiotics or steroids [45].

*C. parapsilosis* is capable of producing a variety of eicosanoids. The prostaglandin profile of *C. parapsilosis* is quite similar to that of *C. albicans*, with PGE_2_ and PGD_2_ being predominantly produced in the presence of arachidonic acid as a sole carbon source. Interestingly, unlike in case of *C. albicans*, the fatty acid desaturase homologous gene *OLE2* does not play a role in their synthesis [46]. Recently, *CPAR2_603600* (homologous of *CaFET3), CPAR2_807710* (homologue of the acyl-coenzyme A oxidase, *ScPOX1-3*) and *CPAR2_800020* (homologue of 3-ketoacyl-CoA thiolase, *ScPOT1*) have been demonstrated to be involved in the generation of fungal eicosanoids in *C. parapsilosis* [47]. LC/MS analysis showed that the disruption of each gene led to a decrease in the production of PGE_2_, PGD_2_ and 15-keto-PGE_2_. The deletion mutant strains of *CPAR2_603600, CPAR2_800020* and *CPAR2_807710* produced less prostaglandin D_2_ (PGD_2_) and also had a significant decrease in PGE_2_ production. However, only the deletion mutant strain of *CPAR2_807710* has a reduction in 15-keto-prostaglandin E_2_ (15-keto-PGE_2_) production. This study also reported the presence of fungal 5-D2-isoprostane in *C. parapsilosis* by LC/MS analysis. The eicosanoid mutant strains were also shown to induce more pro-inflammatory cytokines by human peripheral blood derived macrophages and they were less virulent in a mouse model of systemic candidiasis compared to the wild type strain [47], which indicates the importance of these fungal derived eicosanoids in *C. parapsilosis* virulence and pathogenicity mechanisms.

### Future perspectives

The significance of fungal eicosanoid lipid mediators in pathogenesis is increasingly validated through interesting published and ongoing research, although their biosynthetic pathways and exact function in pathobiology is not yet fully explored. Furthermore, it is also unclear whether these pathogenic yeasts contain specific receptors for their recognition, such as G-protein coupled receptors (GPCRs) in mammalian cells. These immunomodulatory lipid molecules are produced by not only pathogenic yeasts, but also filamentous fungi such as *Aspergillus nidulans* and *A. fumigatus* [48]. Interestingly, eukaryotic parasites, such as *Plasmodium falciparum* [49] and *Trypanosoma brucei* [50], have also recently been shown to produce prostaglandin like compounds. It is possible that eicosanoids secreted by eukaryotic pathogenic organisms can vary in function, and influence microbial growth and maturation, or effect host interactions, by modulating immune responses. Although it remains to be fully elucidated whether microbial eicosanoids are also virulence factors and why the pathogenic fungi belong to a different family evolved with mechanisms for producing these eicosanoid molecules that are structurally similar to the bioactive lipid mediators generated by human hosts.
